# Antidiarrhoea and toxicological evaluation of the leaf extract of *Dissotis rotundifolia *triana (Melastomataceae)

**DOI:** 10.1186/1472-6882-10-71

**Published:** 2010-11-17

**Authors:** Tavs A Abere, Pius E Okoto, Freddy O Agoreyo

**Affiliations:** 1 Department of Pharmacognosy, Faculty of Pharmacy, University of Benin, Benin City. Nigeria; 2 Faculty of Pharmacy, University of Benin, Benin City. Nigeria; 3 Department of Anatomy, College of Medical Sciences, University of Benin, Benin City. Nigeria

## Abstract

**Background:**

The leaves of *Dissotis rotundifolia *are used ethnomedically across Africa without scientific basis or safety concerns. Determination of its phytochemical constituents, antimicrobial activity, effects on the gastrointestinal tract (GIT) as well as toxicological profile will provide supportive scientific evidence in favour of its continous usage.

**Method:**

Chemical and chromatographic tests were employed in phytochemical investigations. Inhibitory activity against clinical strains of *Escherichia coli, Pseudomonas aeruginosa, Staphylococcus aureus *and *Salmonella typhi *were compared with Gentamycin. Our report includes minimum inhibitory concentration (MIC) against the tested organisms. The effect of the ethanol extract on the motility of the GIT in mice using the charcoal plug method and castor oil induced diarrhoea in rats was evaluated. Toxicological evaluation was determined by administering 250 mg/kg and 500 mg/kg of extracts on male Wistar rats for 14 days with normal saline as control. The tissues of the kidneys, liver, heart and testes were examined.

**Results:**

Phytochemical studies revealed the presence of alkaloids, saponin and cardiac glycosides. The crude ethanol extract and fractions inhibited the growth of *E. coli, P. aeruginosa, S. aureus *and *S. typhi *to varying extents. The degree of transition exhibited by the charcoal meal was dose-dependent. In the castor oil induced diarrhoea test, all the doses showed anti-spasmodic effects. The LD50 in mice was above 500 mg/kg body weight. Toxicological evaluation at 500 mg/kg showed increased cytoplasmic eosinophilia and densely stained nuclei of the liver, tubular necrosis of the kidney, presence of ill-defined testes with indistinct cell outlines and no remarkable changes in the heart.

**Conclusion:**

Ethanol extracts of *Dissotis rotundifolia *have demonstrated antimicrobial activity against clinical strains of selected microorganisms. The plant showed potential for application in the treatment of diarrhoea, thereby justifying its usage across Africa. It also demonstrated toxicity in certain organs at the dose of 500 mg/kg, and it will be necessary to fully establish its safety profile.

## Background

Medicinal plants are commonly used in treating and preventing specific ailments and diseases, and are generally considered to play a beneficial role in healthcare. They are already important to the global economy. Demand is steadily increasing not only in developing countries but also in the industrialized nations [1]. World Health Organisation (WHO) estimates that approximately 80% of the developing world's population meets their Primary Healthcare needs through traditional medicine [2]. About 25% of prescription drugs dispensed in the United States contain at least one active ingredient derived from plant material. Some are made from plant extracts while others are synthesized to mimic a natural plant compound [3]. It has been estimated that one out of every three people in the United States had tried at least one form of alternative medicine [4]. A follow up study reported that the number of respondents using alternative therapies increased from 33.8% in 1990 to 42.7% in 1997 [5]. Within the last few decades, many plants have been screened for their biological and pharmacological properties by researchers. These efforts are continually being taken to examine the merits of traditional medicine in the light of modern science with a view aimed at adopting effectively beneficial medical practice and discouraging harmful ones [6].

The genus *Dissotis *comprises of 140 species native to Africa [7]. *Dissotis rotundifolia *Triana, a native of tropical West Africa belongs to the Melastomataceae family [8] and common names include Pinklady (English), Awede (Yoruba) and Ebafo (Bini) [9].

*Dissotis rotundifolia *is a versatile perennial slender creeping herb with prostate or ascending stems up to 40 cm high, rooting at the nodes and producing from seeds and stolon. The leaves are ovate to ovate-lanceolate or suborbicular, 1.5-7.0 cm long, 0.8-4.0 cm wide, 3-nerved, both surfaces sparsely to densely pilose, margins ciliate and somewhat crenuate, apex acute, base truncate to short-attenuate, petioles 0.5-2.5 cm long [10].

In Nigeria, *D. rotundifolia *is used mainly for the treatment of rheumatism and painful swellings. The leaves decoction is used to relieve stomach ache, diarrhea, dysentery, cough, stop abortion, conjunctivitis, circulatory problems and venereal diseases [9]. It is used in East Africa for the treatment of bilharzias [11] and in Cameroun, the leaves are used for dysentery [12]. In tropical Africa, the whole plant is used as a remedy for rheumatism and yaws and as an antihelmintic and in Liberia for diarrhea [13]. Hot water extract of *Dissotis rotundifolia *given orally is used for hookworm infestations [11].

## Methods

### Plant collection and authentication

The leaves of *Dissotis rotundifolia *Triana were collected in Ugbowo area of Benin City, Edo State, Nigeria. The plants were identified and confirmed by the plant curator at the Forest Research Institute of Nigeria (FRIN), Ibadan where voucher specimens were deposited with the number, FIH 107850. The leaves were air-dried and powdered on an electric mill.

### Phytochemical studies

Chemical and Chromatographic tests were employed in the preliminary Phytochemical screening for various secondary metabolites such as Tannins (phenazone; iron complex; formaldehyde and modified iron complex tests), Alkaloids (Mayer's; Dragendorff's, Wagner's and 1% picric acid reagents), Cardiac glycosides (Keller Killiani, Lieberman, Legal and Kedde tests), Saponin glycosides (frothing and haemolysis tests), Anthracene derivatives (Borntrager's test for combined and free anthraquinones) and Cyanogenetic glycosides (sodium picrate paper test) [14-17]. Thin layer chromatography of aqueous ethanol extract containing 10% H_2_SO_4_, shaken with chloroform and free alkaloids precipitated by the addition of excess ammonia and extracted with chloroform, on silica gel-G, activated by heating at 110°C for 30 minutes was developed with the solvent system Methanol : chloroform (3: 7). Chromatoplates were viewed under the UV light and sprayed with Dragendorff's spray reagent and R_f _values calculated. Paper chromatography of the aqueous ethanol extract, using ascending method and Whatman No. 3 mm was developed with the solvent system n-butanol: water: acetic acid (4: 1: 5), examined both in daylight and under UV light at 25 nm, sprayed with ferric chloride until colours developed and R_f _values calculated.

### Antidiarrhoea evaluation

#### Organisms

The organisms used were clinical isolates of *Escherichia coli*, *Pseudomonas aeruginosa*, *Staphylococcus aureus *and *Streptococcus faecalis *obtained from patients in the University of Benin Teaching Hospital, Benin City.

#### Animals

Swiss albino mice of both sexes (26. 87 ± 1. 64 g) and male Wistar rats (207 ± 17. 94 g) were obtained from the Animal House of Pharmacology Department, Faculty of Pharmacy, University of Benin, Benin City. All the animals were kept under standard environmental conditions and were handled according to international protocol for use of animals in experiments. They had free access to food and water i. e. they were fed with standard pellets and tap water *ad libitum*. Permission and approval for animal studies were obtained from the College of Medical Sciences Animal Ethics Committee, University of Benin, Benin City with ADM/F. 22 A/VOL. VIII/349.

#### Intestinal transit test

The mice used for this test were starved for 24 hrs prior to the experiment but allowed access to water. They were divided into 7 groups of 5 each. Mice in groups A, B, C and D were administered with 100, 200, 300 and 400 mg/kg extract, the fifth group 5 ml/kg normal saline (vehicle for the reconstitution of the extract), sixth group 2.5 mg/kg Atropine sulphate (Adrich. Chem. Co. UK) all by the intraperitoneal route. The seventh group received 0.5 mg/kg Carbachol. After 30 minutes, they were fed through the gastric tube with 0.5 ml of freshly prepared charcoal meal (10% charcoal in acacia gum). The mice were sacrificed 20 minutes later by cervical dislocation and gastrointestinal tract removed. The distance travelled by the charcoal meal from the pylorus was measured and expressed as the percentage of the total length of the small intestine extending from the gastropyloric to the ileocaecal junction [18]. The intestinal motility was derived from the equation.

$$\%\text{ motility}=\frac{\text{Distance traveled by the meal}}{\text{Total length of small intestine}}\times 100$$

#### Castor oil induced diarrhoea

Rats for this test were first observed for any wet faeces/droppings (their somewhat rounded or irregular shape, soft consistency and the presence of a brown stain surrounding each faeces on the filter paper). They were easily distinguished from the normal dry faeces which were elongated, regular in shape, hard and did not stain the filter paper. Those that produced wet droppings were not used for the test.

Diarrhoea was induced by oral administration of castor oil (0.5 ml rat ^-1^) to overnight fasted rats. *Dissotis rotundifolia *extract (100 - 400 mg/kg) or vehicle (normal saline) or reference (loperamide) were given intraperitoneally 60 minutes before cathartic administration.

Two hours after dosing with castor oil, the individual rat cages were inspected for the presence of wet faeces, their absence was recorded as a positive result, indicating protection from diarrhoea at that time [19].

#### Preliminary antimicrobial screening

Powdered leaf material (2 kg) of *Dissotis rotundifolia *Triana was extracted with 50% aqueous ethanol at room temperature. After 48 h, the extract was clarified by filtration and evaporated to dryness *in vacuo*. Residues were collected and evaluated against the four test organisms at 100 mg/mL in 50% aqueous methanol.

#### Activity guided fractionation of *Dissotis rotundifolia*

The aqueous ethanolic extract (8 g) of *Dissotis rotundifolia *was partitioned between water and chloroform. The fractions were evaporated to dryness in vacuo. Partitioning using water: ethylacetate and water: butanol were similarly carried out respectively. The resulting fractions were evaluated against the test organisms at 100 mg/mL in aqueous methanol.

#### Antimicrobial test

The well diffusion method was used for the antimicrobial screening against the four test organisms, following standard procedures with nutrient agar as medium.

The minimum inhibitory concentration (MIC), the lowest concentration of a compound that inhibits growth of a microorganism, was determined by the standard two-fold dilution technique using nutrient broth medium [20].

### Toxicological evaluation

#### Acute toxicity test

Swiss albino mice (5 animals per group) were orally treated with the aqueous ethanol extract of *Dissotis rotundifolia *at doses of 1 g/kg, 2 g/kg, 3 g/kg, 4 g/kg and 5 g/kg. The control group received only the vehicle (normal saline). Each group of mice was placed in the test cage for a 30-min habituation period before any oral administration of the drug using an orogastric syringe. The animals were observed for 3 days. Lethality and gross toxicological features were recorded for each group at the end of the period [21]. The animals were further observed for fourteen days.

#### Sub-acute toxicity test

Thirty male Wistar rats were randomly distributed into three groups of ten rats each. The first (A) group served as control and received 5 ml of normal saline (vehicle) while the second (B) and third (C) groups received 250 mg/kg and 500 mg/kg per day of *D. rotundifolia *extract respectively orally for 14 consecutive days. The animals were observed for signs of toxicity (abnormal behaviours, writhing convulsion, mood, motor activity and general body conditions). At the end of 14 days, the rats were sacrificed under chloroform anesthesia. The livers, kidneys, hearts and testes were removed and preserved in 10% formaldehyde. Tissues from each organ were sectioned (6 microns (μ) thick) in paraffin wax and stained with hematoxylin and eosin (H & E) for assessment of tissue morphology [22].

## Results

Phytochemical screening of the leaves of *Dissotis rotundifolia *revealed the presence of alkaloids, tannins, saponins and cardiac glycosides (Table 1). Chromatographic fingerprints were established using Thin-layer and Paper chromatographic techniques (Table 2), detecting with Dragendorff and Ferric chloride for the presence of alkaloids and phenolic compounds respectively.

**Table 1:** Results of preliminary phytochemical screening of *Dissotis rotundifolia *leaves.

Secondary metabolites	
**Alkaloids**	
Dragendorff's reagent	+
Wagner's reagent	+
Mayer's reagent	+
1% picric acid reagent	+
	
**Anthracene derivatives**	
Combined anthraquinones	-
Free anthraquinones	-
	
**Tannins**	
Phenazone test	+
Iron complex test	+
Formaldehyde test	+
Modified iron complex test	+
	
**Saponin glycosides**	
Frothing test	+
Haemolysis test	+
	
**Cardiac glycosides**	
Keller killiani test	+
Lieberman's test	+
Legal test	+
Kedde test	+
	
**Cyanogenetic glycosides**	
Sodium picrate test paper	-

Key:

- = absent; + = present

**Table 2:** Chromatographic results

Thin layer chromatography						
**Extract**	**Solvent System**	**No. of spots**	**Colour in Daylight**	**Colour in UV**	**Colour after spraying with Dragendorff**	**R**_**f **_**value**

Aqueous ethanol	Methanol: chloroform (3 : 7)	2	Colourless	Light green fluorescent	Reddish brown	0.78
			colourless	Light green fluorescent	Reddish brown	0.80

**Paper chromatography**						

**Extract**	**Solvent System**	**No. of spots**	**Colour in daylight**	**Colour in UV**	**Colour after spraying with ferric chloride**	**R**_**f **_**value**

Aqueous ethanol	N-butanol: water: acetic acid (4: 1: 5)	1	Colourless	green fluorescent	Blue black colour	0.21

Aqueous ethanol extract of *D. rotundifolia *showed antimicrobial activity against the tested organisms (Table 3). The activities of the various fractions as well as the minimum inhibitory concentration (MIC) of the aqueous ethanol extract are presented in Tables 4 and 5 respectively. The effects of the ethanol extract on GIT motility (Table 6) showed a reduction in percentage movement of charcoal plug in a dose dependent manner. In the castor oil induced diarrhea experiment (Table 7), the concentrations of the ethanol extract used as well as the reference standard, Loperamide did not produce any wet faeces.

**Table 3:** Antimicrobial screening of *Dissotis rotundifolia *leaves at 100 mg/mL, in methanol: water (1: 1).

Test organism	Aq. ethanol extract of *D. rotundifolia*	Gentamycin (10 μg/ml)	Solvent Aq. methanol
	(mm)	(mm)	(mm)
*Escherichia coli*	8	13	0
*Pseudomonas aeruginosa*	11	15	0
*Staphylococcus aureus*	8	14	0
*Salmonella typhi*	9	14	0

The diameter recorded is the diameter of the zone of inhibition less the diameter of the cup (8 mm).

Values are mean (n = 5)

**Table 4:** Antimicrobial screening of three fractions of *D. rotundifolia *extract at 100 mg/Ml, in methanol: water (1: 1).

Test organism	Chloroform fraction	Ethylacetate fraction	Butanol fraction	Aqueous fraction
	(mm)	(mm)	(mm)	(mm)
*Escherichia coli*	-	2	5	-
*Pseudomonas aeruginosa*	-	-	7	3
*Staphylococcus aureus*	1	2	5	2
*Salmonella typhi*	1	1	4	-

The diameter recorded is the diameter of the zone of inhibition less the diameter of the cup (8 mm).

Values are mean (n = 5)

**Table 5:** Minimum inhibitory concentration MIC (μg/mL) of *D. rotundifolia *leaves.

Test organism	MIC (μg/mL) of aq. ethanol extract of *D. Rotundifolia*
*Escherichia coli*	50
*Pseudomonas aeruginosa*	25
*Staphylococcus aureus*	50
*Salmonella typhi*	50

Values are mean (n = 5)

**Table 6:** Effects of ethanol extract of *D. rotundifolia *on GIT motility

Group	% movement of charcoal plug (mean ± SEM)
Normal saline	31. 38 ± 6. 03
2. 5 mg/kg of Atropine	25. 30 ± 3. 56
0. 5 mg/kg of Carbachol	68. 49 ± 10. 34
100 mg/kg of *D. rotundifolia*	14. 35 ± 1. 10
200 mg/kg of *D. rotundifolia*	5. 45 ± 2. 99
300 mg/kg of *D. rotundifolia*	2. 64 ± 0. 35
400 mg/kg of *D. rotundifolia*	1. 90 ± 0. 20

Values are mean + SEM (n = 5)

**Table 7:** Effects of ethanol extract of *D. rotundifolia *on Castor oil induced diarrhoea

Group	Presence of wet faeces
Normal saline	+
0. 03 mg/kg of Loperamide	-
100 mg/kg of *D. rotundifolia*	-
200 mg/kg of *D. rotundifolia*	-
300 mg/kg of *D. rotundifolia*	-
400 mg/kg of *D. rotundifolia*	-

Key: - = absence; + = presence

### Acute toxicity profile

In the acute toxicity study, the aqueous extract of *D. rotundifolia *did not produce any mortality up to the oral dose level of 5 g/kg body weight in mice, hence the LD50 was indeterminable. There were no significant changes in the behaviour in relation to the posture, diarrhoea, mood and motor activity. The animals did not convulse nor exhibited writhings. However, in the sub-acute toxicity test (Table 8), there were clear evidence of histopathological effects in most of the organs, especially at the dose of 500 mg/kg.

**Table 8:** Histological effects of the administration of ethanol extracts of *D*. *rotundifolia *on rats.

Dosage of administration	Histological effect on liver tissues	Histological effect on kidney tissues	Histological effect on Heart tissues	Histological effect on the testes
0. 9 ml of normal saline	Tissues appeared normal. No evidence of tissue necrosis	Tissues appeared normal. No evidence of tissue necrosis	Tissues appeared normal. No evidence of tissue necrosis	Tissues appeared normal. No evidence of tissue necrosis

250 mg/kg of *D. rotundifolia*	Densely stained nuclei, increased congestion of the vessels, all of which are evidence of tissue necrosis	Tissues appeared normal. No evidence of tissue necrosis	Tissues appeared normal. No evidence of tissue necrosis	Tissues appeared normal. No evidence of tissue necrosis

500 mg/kg of *D. rotundifolia*	There was increased cytoplasmic eosinophilia and densely stained nuclei. There was also increased congestion of the vessels. These are evidence of liver cell damage.	Showed tubular necrosis and degenerative changes in the tubules	Tissues appeared normal. No evidence of tissue necrosis	Presence of ill defined testes with indistinct cell outlines.

## Discussion

The need for phytochemical screening has become imperative, since many plants accumulate biologically active, complex organic chemicals (secondary plant metabolites) in their tissues. *Dissotis rotundifolia *revealed the presence of alkaloids, saponins and cardiac glycosides. Typical alkaloids often have marked pharmacological effects when administered to man and other animals, thus their presence is of particular interest [17]. The pharmacological effectiveness of glycosides is dependent on the aglycones, but the sugars render the compounds more soluble and increase the power of fixation of the glycosides. Plants containing alkaloids and/or glycosides have been known to possess antidiarrhoeal activities, amongst which are *Ageratum conyzoides *and *Vernonia cinerea *[23]. It could therefore be suggested that the secondary plant metabolites present in *D. rotundifolia *are responsible for the biological activities observed.

Most diarrhoeas are known to be infective in origin and there is therefore a role for anti-infective agents in the management of such diarrhoeas [23]. Among other possible causative microorganisms implicated in diarrhoea, the role of enteroaggregative *Escherichia coli *has been reported [24]. The aqueous ethanol extract alongside the partitioned fractions were subjected to antibacterial test at 100 mg/ml so as to locate the fraction with the highest activity. The results indicated that the aqueous ethanol extract compared favourably with Gentamycin for the gram-ve and gram+ve organisms employed. The results also indicated that the butanol fraction was the most active.

Another common observable feature in diarrhoea cases is hypermotility of the gut. Reduction of this motility has been achieved by inclusion of anti-spasmodic agents in preparations intended for use in the treatment of diarrhoea. The effects of graded doses of *D. rotundifolia *extract on intestinal transit using freshly prepared charcoal meal was examined using atropine and carbachol as reference drugs (both have known effects on the GI tract). The % age movement of the charcoal plug was reduced by the extract (P < 0. 05) in a dose dependent manner. In the castor oil induced diarrhoea experiment, the extracts exhibited the same characteristics as the reference, Loperamide by non-production of wet faeces. Inhibition of the intestinal motility and non-production wet faeces could be useful actions in the treatment of diarrhoea.

Toxicity potential of a new drug is assessed in the light of the purpose for which it is to be used and the period over which it will be administered. For example, vomiting as a toxic effect will disqualify a drug which is being develop for a minor complaint, but will be accepted in a drug to be used for the treatment of cancer and other life-threatening conditions. Also a toxic effect which develops after prolong administration may not disqualify a drug intended for acute condition or for only occasional administration. It has been stated that all substances have potential toxicity and that all herbs can, therefore be harmful, but most would have to be ingested in impossible amounts to cause harm [25].

The primary determinant of the safety of a substance is the dose. The dangers of herbal drug therapy these days are all too often due to lack of stringent assurance rather than to the pharmacological activity of the herbal ingredients themselves. Toxicological studies for all herbal medicines including the determination of their median lethal dose (LD50) and other such parameters essential for a proper dosage are desirable and necessary. If there is the suspected need for more detailed studies, such herbal medicines may be subjected to sub-acute and chronic toxicity tests.

The purpose of an acute toxicity test is to determine the nature and extent of the adverse reactions to a single dose or an overdose of the drug [25]. Increasing doses of aqueous ethanol extracts of *D. rotundifolia *up to 5 g/kg administered to mice per os were not lethal, so that the LD50 could not be determined. It is an indication of the low toxicity of the extracts [21], therefore *D. rotundifolia *can be said to be relatively safe and may be used for the treatment of diarrhoea. It has been realized that a high degree of precision may not be necessary to compare toxicity [26]. Therefore, the LD50 is now an approximate value estimated from the smallest number of animals possible. The dose is used to calculate the initial dose to be tried in man during the clinical trials. It has been suggested that a simple *limit *test, aimed at determining the effect on animals, of the largest dose likely to be administered to a human being is often enough [27].

The general purpose of the sub-acute toxicity tests is to determine the organs that are likely to be susceptible to toxicity by the herbal medicines. Histopathological effects of the liver of rats administered 250 mg/kg and 500 mg/kg per day of the aqueous ethanol extracts of *D. rotundifolia *showed increased cytoplasmic eosinophilia and densely stained nuclei compared to the control. There was also increased congestion of the vessels. These are evidence of liver cell damage. The kidneys of the rats administered *D. rotundifolia *showed tubular necrosis and degenerative changes in the tubules. The sections of the hearts administered *D. rotundifolia *and normal saline were not significantly affected. There were no marked adverse alterations or degeneration of tissues since these vital organs showed normal architectures suggesting no morphological disruptions as compared with the control group.

## Conclusion

Herbal medicines can gain the confidence of orthodox health practitioners when there are scientifically established proofs of their claimed efficacies. On the basis of the overall results from our investigations, the use of *D. rotundifolia *in the treatment of diarrhoea, veneral diseases, dysentery and relief of stomach ache is justified. Extracts of *D. rotundifolia *have in this work been found to possess antimicrobial and antispasmodic activities which makes it good candidate for further works in diarrhoea management. It is however advised that herbal practitioners should be aware of the likely effects that may occur, especially on prolonged usage.

## Competing interests

The authors declare that they have no competing interests.

## Authors' contributions

TA carried out the plant preparations, Antimicrobial and phytochemical investigations, PO carried out the Antidiarrhoea experiments while FA carried out the Toxicity evaluation. All authors read and approved the final manuscript.

## Pre-publication history

The pre-publication history for this paper can be accessed here:

http://www.biomedcentral.com/1472-6882/10/71/prepub
